# Differentiating effective salvage from ineffective delayed compensation in anterior circulation stroke: a dynamic quantitative collateral index

**DOI:** 10.3389/fmed.2026.1862145

**Published:** 2026-06-18

**Authors:** Zhong Zheng, Ailixier Tayier, Dayu Wu, Weiping Lu, Ziying Wang, Tong Hou, Hongqing Zhu, Ying Wang, Bingcang Huang

**Affiliations:** 1 Postgraduate Training Base at Shanghai Gongli Hospital, Ningxia Medical University, Shanghai, China; 2 Department of Neurology, Gongli Hospital of Shanghai Pudong New Area, Shanghai, China; 3 Department of Radiology, Gongli Hospital of Shanghai Pudong New Area, Shanghai, China; 4 School of Information Science and Engineering, East China University of Science and Technology, Shanghai, China; 5 Shanghai Health Commission Key Lab of Artificial Intelligence (AI)-Based Management of Inflammation and Chronic Diseases, Sino-French Cooperative Central Lab, Gongli Hospital of Shanghai Pudong New Area, Shanghai, China

**Keywords:** collateral circulation, computed tomography perfusion, early neurological recovery, hemodynamics, ischemic stroke

## Abstract

**Objectives:**

To investigate a deconvolution-free Dynamic Quantitative Collateral Approach based on 4D-CTA reconstructed from CTP source data, and to evaluate its prognostic value for Early Neurological Recovery (ENR) in acute anterior circulation large vessel occlusion (LVO) by differentiating effective tissue salvage from ineffective delayed perfusion.

**Methods:**

In this retrospective study of 94 patients with anterior circulation LVO, the Rapid Compensation Index (RCI), Late Compensation Index (LCI), and Total Compensation Index (TCI) were calculated based on Vessel Volume Deficit (VVD) derived from 4D-CTA reconstructed from CTP source data. Logistic regression, ROC analysis, and Akaike Information Criterion (AIC) were utilized to compare the quantitative collateral indices against visual scores (Tan, rLMC, Menon) for predicting ENR (7-day ΔNIHSS ≥4). A predictive nomogram was constructed and evaluated for clinical net benefit.

**Results:**

RCI significantly predicted recovery, whereas LCI showed limited utility, objectively reflecting delayed collateral filling. TCI independently predicted ENR with high discrimination (AUC = 0.858), significantly outperforming the Tan score (AUC = 0.810, *p* = 0.012) and equating to the expert multiphase Menon score (*p* = 0.313). TCI demonstrated superior model fit (AIC = 92.02) and a strong inverse correlation with infarct core volume (*ρ* = −0.85). The TCI-integrated nomogram exhibited excellent calibration and clinical net benefit.

**Conclusion:**

This dynamic approach provides an objective, deconvolution-free assessment of hemodynamic efficiency. By physiologically distinguishing functional reperfusion from ineffective delayed compensation, it demonstrates prognostic accuracy comparable to expert consensus, offering a robust tool for acute stroke triage.

## 1 Introduction

Collateral circulation status determines penumbral survival in acute large vessel occlusion (LVO) and guides treatment decision-making (1, 2). However, accurately quantifying dynamic collateral flow remains a key clinical challenge. Current semi-quantitative clinical tools [e.g., Tan and regional Leptomeningeal Collateral (rLMC) scores] depend on static visual inspection, which carries substantial inter-observer variability and fails to capture the temporal efficiency of hemodynamic compensation (3–5). While Standard computed tomography perfusion (CTP) generates reliable quantitative perfusion maps and captures temporal dynamics (6), the traditional deconvolution algorithms underlying this technique are highly sensitive to severe tracer delay and low-flow states (7, 8). Such limitations can compromise volumetric measurement accuracy in critically ischemic conditions, frequently overestimating the ischemic core (9, 10). In acute anterior circulation large vessel occlusion (LVO), retrograde collateral flow introduces inherent tracer delay and bolus dispersion within the ischemic territory. These complex hemodynamic alterations deviate from the ideal conditions assumed by standard deconvolution algorithms, which might lead to the artificial depression of relative cerebral blood flow (rCBF) thresholds, inadvertently contributing to the overestimation of the ischemic core (the “ghost core” phenomenon) (11).

Time-resolved 4D-CTA, utilizing multiphase Maximum Intensity Projections (e.g., early, peak, and late phases), has been shown to provide a more comprehensive assessment of dynamic collateral filling than static imaging (12, 13). However, current time-resolved approaches still depend on semi-quantitative visual grading, leaving them susceptible to inter-observer variability and prognostic ceiling effects. In this study, we employed a modified deconvolution-free image-processing approach to extract Vessel Volume Deficit (VVD) across arterial, venous, and late venous phases. This algorithm relies on voxel-wise mirror-subtraction to quantify peak-density attenuation differences based on established multiphase hemodynamic principles. By evaluating physical density gradients rather than performing time-dependent parametric curve-fitting, this approach might bypass standard kinetic modeling biases and minimizes susceptibility to tracer delay- and dispersion-induced artifacts.

While raw VVD offers precise anatomical measurements, isolated volumetric data cannot reliably distinguish effective tissue salvage from ineffective delayed compensation. We therefore developed the Dynamic Quantitative Collateral Approach based on time-resolved VVD, introducing the Rapid Compensation Index (RCI), Late Compensation Index (LCI), and Total Compensation Index (TCI). We hypothesize that the quantitative collateral indices can characterize collateral perfusion across all time phases and distinguish effective early compensatory flow from ineffective delayed collateral filling. By evaluating the quantitative collateral indices against early neurological recovery (ENR) (14)—*a direct clinical indicator of acute tissue salvage*—we aimed to determine if this dynamic approach could serve as an objective alternative to expert multiphase visual assessment (e.g., the Menon score) (15).

## 2 Methods

### 2.1 Clinical data

We retrospectively analyzed patients with acute ischemic stroke (AIS) treated at the Stroke Center of Shanghai Pudong New Area Gongli Hospital between February 2021 and July 2025. The study protocol was approved by the hospital ethics committee, with a waiver for informed consent.

Inclusion criteria were: (1) age > 18 years; (2) time from onset to admission < 24 h; (3) completion of a one-stop CT protocol (NCCT + 4D-CTA + CTP) (16) confirming unilateral anterior circulation LVO (distal ICA, MCA-M1, or M2 segments); (4) achieving successful and complete recanalization, defined strictly as a modified Treatment in Cerebral Ischemia (mTICI) grade of 3, for all patients undergoing endovascular thrombectomy (EVT); and (5)availability of complete baseline and 7-day follow-up data. Exclusion criteria included intracranial hemorrhage, bilateral infarction or prior large territorial infarction affecting the symptomatic side (interfering with perfusion analysis), severe dysfunction of the heart, liver, kidney, or hematological system, or poor image quality precluding post-processing.

Demographic characteristics, vascular risk factors, stroke etiology (TOAST classification), occlusion site (ICA, MCA-M1, MCA-M2), and crucial time metrics (onset-to-imaging and onset-to-recanalization times) were systematically collected. The primary outcome was Early Neurological Recovery (ENR), defined as a decrease of ≥ 4 points in the NIHSS score from the baseline admission to the 7-day follow-up. This endpoint was selected to specifically assess the acute therapeutic effect of collateral compensation (14). To account for mortality as a competing risk, patients who died within the 7-day follow-up period were assigned a maximum NIHSS score of 42. This imputation ensures that fatal outcomes result in a strongly negative ΔNIHSS. Patients were stratified into a Significant Improvement Group (ΔNIHSS ≥ 4) and a No Significant Improvement Group (ΔNIHSS < 4).

### 2.2 Scanning protocol

All patients underwent scanning using a 128-slice dual-source CT (Somatom Definition FLASH, Siemens Healthineers, Erlangen, Germany). The protocol included NCCT (120 kV, 350 mAs, 5 mm slice thickness) followed by 4D CTA-CTP using shuttle-mode technology. The dynamic scan (80 kV, 90 mAs) commenced 4 s after the injection of 50 mL iodixanol (320 mg I/mL) via the antecubital vein at 5 mL/s (followed by a 20 mL saline flush), covering 160 mm with 30 whole-brain volume sequences acquired over 39 s.

### 2.3 Image post-processing

#### 2.3.1 4D-CTA collateral quantification (VVD analysis)

The Vessel Volume Deficit (VVD) was extracted using a commercial neuroimaging software (CerebralDoc, version 1.0.8, Shukun Technology, Beijing, China), which has been approved for clinical use. This software reconstructs 4D-CTA from dynamic CTP source images and extracts VVD without relying on traditional deconvolution algorithms. Specifically, VVD is volumetrically quantified by calculating the volume of poorly opacified or unperfused vessels in the affected hemisphere. This is achieved via automated contralateral mirror comparison on phase-specific Maximum Intensity Projections (MIPs) performed by the software. To ensure objective phase selection across the 30-frame CTP time series, the software utilizes automated Time-Density Curve (TDC) tracking. The arterial phase is defined by the peak TDC of the contralateral non-occluded Middle Cerebral Artery (MCA), the venous phase by the peak TDC of the Superior Sagittal Sinus, and the late venous phase is defined as the frame acquired 8 s subsequent to the venous peak (17). This automated segmentation yields absolute deficit volumes at three critical hemodynamic phases:

*Arterial Phase Deficit (VVD_a_)*: Quantified at time of peak arterial opacification on the TDC of the contralateral arterial system. This reflects the instantaneous flow cessation caused by proximal occlusion, representing the maximum initial ischemic burden.

*Venous Phase Deficit (VVD_v_)*: Quantified at the time of peak opacification on the TDC of the superior sagittal sinus. This volume is typically reduced relative to *VVD*_a_, reflecting the attenuation of the initial ischemic burden via partial collateral compensation.

*Late Venous Phase Deficit (VVD_d_)*: Quantified at the late venous phase (17). This persistent unperfused volume delineates tissue devoid of effective collateral compensation, serving as an imaging surrogate for the ischemic core.

#### 2.3.2 CTP parameter analysis

Perfusion maps were generated using automated CTP analysis software (F-STROKE, NaoXi Intelligent Technology, Shanghai, China). Regions of interest were defined based on established thresholds: the ischemic core was identified as the volume with rCBF < 30%, while the total hypoperfused volume was defined as tissue with Tmax > 6 s. The ischemic penumbra (mismatch volume) was subsequently calculated by subtracting the ischemic core volume from the total hypoperfused volume (18). Additionally, the Hypoperfusion Intensity Ratio (HIR) was calculated as the volume with Tmax > 10s divided by the volume with Tmax > 6 s (19), and the CBV Index was derived from the mean CBV in the penumbra normalized to the mean CBV in the contralateral mirrored region (20).

### 2.4 Collateral assessment

#### 2.4.1 Visual semi-quantitative assessment

Two senior neuroradiologists blinded to clinical information independently reviewed images; disagreements were resolved by consensus. The following three standard scoring systems were used:

*Tan Score*: Based on single-phase CTA, assessing distal MCA vascular filling (0–3) (3).

*rLMC Score*: Based on single-phase CTA, scoring 10 anatomical regions in the MCA and ACA territories (0–20) (4).

*Menon Score*: Based on multiphase CTA, incorporating filling extent and delay time (0–5) (15).

#### 2.4.2 Construction of quantitative collateral indices

**Figure 1:**
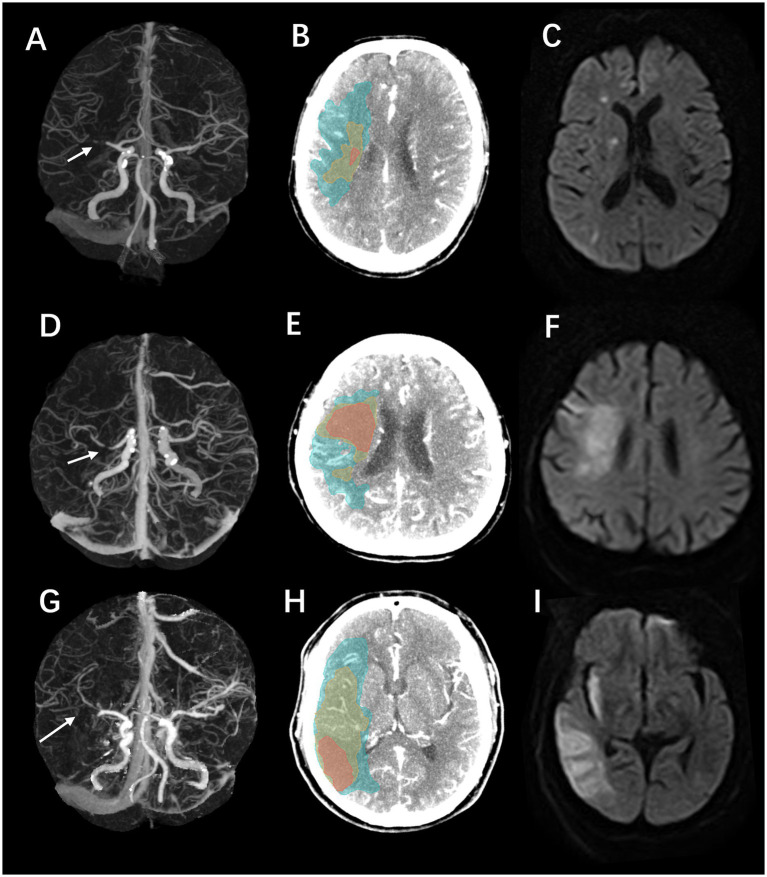
Representative cases illustrating the three distinct collateral hemodynamic phenotypes and their corresponding dynamic quantitative collateral indices. All three patients presented with acute MCA M1 occlusion and identical intermediate visual collateral scores (Tan = 2) but experienced divergent clinical outcomes. The automated dynamic VVD maps display the initial arterial deficit (teal overlay, VVDa), the residual deficit in the venous phase (yellow overlay, VVDv), and the final uncompensated deficit in the late venous phase (red overlay, VVDd). **(A–C)** Scenario A: Effective Collateral Compensation (Significant Improvement, ΔNIHSS = 12). Visual scores were intermediate to high (Tan = 2, rLMC = 13). The large initial arterial deficit resolves rapidly, leaving only a minimal residual deficit in the late venous phase. Quantitative metrics: VVDa = 126.60 mL, VVDv = 12.13 mL, VVDd = 0.38 mL; RCI = 90%, LCI = 96%, TCI = 99%. Follow-up DWI **(C)** confirms excellent tissue salvage. **(D–F)** Scenario B: Minimal Collateral Compensation (No Significant Improvement, ΔNIHSS = −7). Visual scores were comparable to the patient above (Tan = 2, rLMC = 10). The deficit remains extensive throughout all scan phases, indicating near-absent hemodynamic compensation. Quantitative metrics: VVDa = 143.56 mL, VVDv = 85.67 mL, VVDd = 60.30 mL; RCI = 40%, LCI = 29%, TCI = 58%. Follow-up DWI **(F)** demonstrates an extensive territorial infarction. **(G–I)** Scenario C: Ineffective Delayed Collateral Compensation (No Significant Improvement, ΔNIHSS = −10). Visual scores were similarly intermediate (Tan = 2, rLMC = 10). There is a limited reduction in vascular deficit in the early arterial-to-venous phase, followed by a notable reduction in the late venous phase. Quantitative metrics: VVDa = 326.06 mL, VVDv = 217.40 mL, VVDd = 65.22 mL; RCI = 33%, LCI = 70%, TCI = 80%. Despite late-phase contrast arrival resulting in a seemingly acceptable TCI, the delayed collateral flow missed the critical window of tissue viability, failing to halt the evolution of an extensive infarct core **(I)**. MCA, Middle Cerebral Artery; CTA, Computed Tomography Angiography; MIP, Maximum Intensity Projection; rLMC, regional Leptomeningeal Collateral; VVD, Vessel Volume Deficit; TCI, Total Compensation Index; DWI, Diffusion-Weighted Imaging; NIHSS, National Institutes of Health Stroke Scale.

To quantify dynamic collateral efficiency, we constructed the quantitative collateral indices based on Vessel Volume Deficit (VVD): Arterial Phase Deficit (VVDa), Venous Phase Deficit (VVDv), and Late Venous Phase Deficit (VVDd). The clinical translation of these indices across distinct hemodynamic phenotypes is illustrated in Figure 1. Given that absolute ischemic volumes are directly influenced by individual brain volume and the proximal occlusion site (such as the ICA vs. MCA), raw volumetric data should not be compared directly across a heterogeneous cohort. We therefore converted these absolute deficits into relative percentage changes to normalize the baseline stroke severity and standardize the measurement of collateral efficiency.

*Rapid Compensation Index (RCI)*: This index reflects early rapid filling efficiency from the arterial to the venous phase. A higher RCI indicates a robust ability of collateral flow to rapidly reach and salvage ischemic tissue in the early stage.

$$\mathrm{RCI}=\frac{\mathrm{VV}D_{a}-\mathrm{VV}D_{v}}{\mathrm{VV}D_{a}}\times 100\%$$

*Late Compensation Index (LCI)*: This index quantifies the delayed collateral filling from the venous to the late venous phase. A higher LCI indicates pronounced contrast stasis and slow-flow dynamics, serving as a quantitative proxy to evaluate hemodynamically ineffective delayed filling within the ischemic bed.

$$\mathrm{LCI}=\frac{\mathrm{VV}D_{v}-\mathrm{VV}D_{d}}{\mathrm{VV}D_{v}}\times 100\%$$

*Total Compensation Index (TCI)*: This index reflects the overall compensation range of collateral circulation throughout the entire scanning cycle. A higher TCI is predictive of a smaller final infarct core volume.

$$\mathrm{TCI}=\frac{\mathrm{VV}D_{a}-\mathrm{VV}D_{d}}{\mathrm{VV}D_{a}}\times 100\%$$

## 3 Statistical analysis

Statistical analysis was performed using R software (version 4.2.0; packages rms, pROC, and rmda) and SPSS (version 26.0, IBM Corp, Armonk, NY). Normally distributed continuous variables are presented as mean ± standard deviation (SD), and non-normally distributed variables as median (interquartile range, IQR); categorical variables are described as frequencies (percentages). All tests were two-sided, with P<0.05 considered statistically significant.

Group differences between the “Significant Improvement” and “No Significant Improvement” groups were analyzed using the independent samples t -test or Mann–Whitney U test for continuous variables, and the $\chi^{2}$ test or Fisher’s exact test for categorical variables. To evaluate the criterion validity of the novel quantitative indices, we calculated Spearman rank correlation coefficients ( ρ ) to analyze their association with traditional semi-quantitative scores (Tan, Menon, rLMC) in addition to other key imaging parameters (e.g., infarct volume, penumbra volume).

For prediction modeling, Early Neurological Improvement ( ΔNIHSS≥4 ) served as the binary outcome. First, Receiver Operating Characteristic (ROC) curve analysis was used to calculate the Area Under the Curve (AUC) for each imaging metric. Subsequently, multivariable binary logistic regression models were constructed using a hierarchical approach to estimate the Odds Ratios (ORs) and 95% Confidence Intervals (CIs). A clinical “Base Model” was first established incorporating core baseline confounders: Age, Admission NIHSS, Hypertension, HbA1c, and Occlusion Site. To ensure model stability and prevent overfitting given the limited number of outcome events (*N* = 31 in the improvement cohort), variables lacking a significant univariate association with the outcome, such as treatment modality (*p* = 0.29), were not forced into the multivariable model. Following the Base Model, quantitative imaging indices and hemodynamic parameters were individually added to evaluate their independent predictive value. The DeLong test was used to compare AUC changes to evaluate the incremental predictive value of the novel indices (21).

To facilitate individualized risk stratification, a visual nomogram was developed based on the final multivariable logistic regression model. The model’s discriminative performance was quantified using the concordance index (C-index), and calibration was assessed via calibration plots comparing predicted probabilities with observed outcomes. Given the potential for overfitting in finite samples, we performed internal validation using the Bootstrap method with 1,000 resamples (22). The mean optimism was calculated to derive the bias-corrected C-index. Furthermore, Decision Curve Analysis (DCA) was employed to evaluate the clinical net benefit of the TCI-integrated model across a range of reasonable threshold probabilities.

## 4 Results

### 4.1 Baseline clinical and imaging characteristics

The study included 94 AIS patients (median age 73.0 years; 57.4% male). Based on early neurological recovery, 31 patients (33.0%) were classified into the Significant Improvement Group (ENI+), and 63 (67.0%) into the No Significant Improvement Group (ENI-).

There were no statistically significant differences between groups regarding age, admission NIHSS score, or treatment modality (IVT/EVT) (all *p* 0.05) (Table 1). However, the Significant Improvement Group had a significantly higher proportion of males (74% vs. 49%, *p* = 0.037) and better hypertension management, with a lower rate of uncontrolled hypertension (3.2% vs. 24%, *p* = 0.031). Additionally, the No Significant Improvement Group had higher baseline HbA1c (median 6.0% vs. 5.6%, *p* = 0.032) and lactate levels (*p* = 0.042). Homocysteine levels were found to be higher in the significant improvement group (median 13.3 vs. 12.5, *p* = 0.013). Similarly, stroke etiology (TOAST classification), onset-to-imaging time (median 92.5 min, *p* = 0.10), and onset-to-recanalization time in the EVT subgroup (*p* = 0.077) showed no significant differences between the two groups. However, the distribution of occlusion sites varied significantly (*p* = 0.025), reflecting anatomical heterogeneity in baseline ischemic risk.

**Table 1:** Baseline clinical and imaging characteristics.

Variable	Overall *N* = 94	No significant improvement *N* = 63	Significant improvement *N* = 31	*p*-value^1^
Age, (y) Median (Q1, Q3)	73.0 (64.0, 82.0)	73.0 (64.0, 83.0)	73.0 (64.0, 79.0)	0.49
Sex, *n* (%)				**0.037**
Female	40 (43%)	32 (51%)	8 (26%)	
Male	54 (57%)	31 (49%)	23 (74%)	
Smoking, n (%)	35 (37%)	21 (33%)	14 (45%)	0.37
Diabetes mellitus, *n* (%)	29 (31%)	22 (35%)	7 (23%)	0.33
Atrial fibrillation, *n* (%)	38 (40%)	27 (43%)	11 (35%)	0.64
Admission NIHSS, (Score) median (Q1, Q3)	12.0 (9.0, 15.0)	12.0 (8.0, 15.0)	12.0 (10.0, 16.0)	0.22
TOAST classification, *n* (%)				0.35
LAA	52 (55%)	33 (52%)	19 (61%)	
CE	36 (38%)	27 (43%)	9 (29%)	
SOE	6 (6.4%)	3 (4.8%)	3 (9.7%)	
Occlusion site, *n* (%)				**0.025**
ICA	48 (51%)	26 (41%)	22 (71%)	
MCA1	35 (37%)	28 (44%)	7 (23%)	
MCA2	11 (12%)	9 (14%)	2 (6.5%)	
Onset-to-imaging time (min), median (Q1, Q3)	92.5 (64.0, 174.0)	99.0 (67.0, 196.0)	91.0 (56.0, 137.0)	0.10
EVT subgroup characteristics, n (%)	(*N* = 82)	(*n* = 54)	(*n* = 28)	
Onset-to-recanalization time (min), median (Q1, Q3)	350.0 (275.0, 470.0)	370.0 (300.0, 490.0)	325.0 (250.0, 425.0)	0.077
Treatment, *n* (%)				0.29
IVT	11 (12%)	9 (14%)	2 (6.5%)	
EVT	44 (47%)	31 (49%)	13 (42%)	
IVT + EVT	39 (41%)	23 (37%)	16 (52%)	
Hypertension, *n* (%)				**0.031**
No hypertension	25 (27%)	17 (27%)	8 (26%)	
Controlled	53 (56%)	31 (49%)	22 (71%)	
Uncontrolled	16 (17%)	15 (24%)	1 (3.2%)	
Coronary heart disease, n (%)	23 (24%)	16 (25%)	7 (23%)	0.97
RCI, (%) median (Q1, Q3)	76.3 (58.3, 90.3)	66.1 (53.4, 86.8)	84.7 (74.6, 94.7)	**<0.001**
TCI, (%) median (Q1, Q3)	91.6 (72.7, 98.8)	83.4 (62.9, 98.1)	96.0 (91.7, 99.4)	**<0.001**
LCI, (%) median (Q1, Q3)	55.0 (23.9, 77.7)	36.7 (14.6, 68.4)	71.3 (51.8, 86.6)	**0.003**
Tan, (score) median (Q1, Q3)	2.0 (1.0, 2.0)	2.0 (1.0, 2.0)	2.0 (2.0, 3.0)	**<0.001**
rLMC, (score) median (Q1, Q3)	13.0 (9.0, 17.0)	11.0 (8.0, 16.0)	16.0 (14.0, 17.0)	**<0.001**
Menon, (score) median (Q1, Q3)	3.5 (2.0, 4.0)	3.0 (2.0, 4.0)	4.0 (4.0, 4.0)	**<0.001**
CBVIndex, median (Q1, Q3)	0.7 (0.5, 0.8)	0.5 (0.4, 0.7)	0.7 (0.7, 0.9)	**<0.001**
HIR, median (Q1, Q3)	0.3 (0.2, 0.5)	0.4 (0.2, 0.6)	0.3 (0.1, 0.5)	0.12
IC_volume, (mL) median (Q1, Q3)	23.7 (4.2, 97.5)	39.6 (4.4, 111.2)	15.0 (2.8, 31.9)	**0.005**
IP_volume, (mL) median (Q1, Q3)	147.8 (88.0, 218.7)	147.5 (88.0, 221.0)	149.3 (85.5, 208.8)	0.81
mismatch_volume, (mL) median (Q1, Q3)	93.1 (47.7, 135.5)	86.0 (32.4, 122.7)	112.5 (66.8, 150.7)	0.050
Lactate, (mmol/L) median (Q1, Q3)	1.2 (1.0, 1.8)	1.3 (1.0, 1.9)	1.0 (0.9, 1.6)	**0.042**
HbA1c, (%) median (Q1, Q3)	5.9 (5.5, 7.1)	6.0 (5.6, 7.5)	5.6 (5.3, 6.7)	**0.032**
Homocysteine, (μmol/L) median (Q1, Q3)	12.5 (10.4, 16.1)	12.5 (10.0, 13.2)	13.3 (11.5, 21.2)	**0.013**
BNP, (pg/mL) median (Q1, Q3)	97.6 (19.3, 250.0)	112.0 (22.5, 286.0)	63.2 (11.2, 181.0)	0.29
Total cholesterol, (mmol/L) median (Q1, Q3)	4.0 (3.2, 4.5)	4.0 (3.5, 4.5)	3.8 (3.0, 4.2)	0.083

Bold values indicate statistical significance (*P* < 0.05). Wilcoxon rank sum test; Pearson’s Chi-squared test. IVT, Intravenous Thrombolysis; EVT, Endovascular Thrombectomy; RCI, Rapid Compensation Index; TCI, Total Compensation Index; LCI, Late Compensation Index; rLMC, regional Leptomeningeal Collateral; CBV, Cerebral Blood Volume; HIR, Hypoperfusion Intensity Ratio; IC, Infarct Core; IP, Ischemic Penumbra; HbA1c, Glycated Hemoglobin; BNP, Brain Natriuretic Peptide.

Regarding imaging characteristics, the Significant Improvement Group exhibited superior collateral status and less tissue injury. Compared to the non-improvement group, RCI (median 84.7% vs. 66.1%, *p* <0.001) and TCI (median 96.0% vs. 83.4%, *p* <0.001) were significantly higher. This finding was consistent with traditional manual scores (Menon, rLMC, Tan; all *p* <0.001). Furthermore, the Significant Improvement Group had significantly smaller infarct core volumes (15.0 mL vs. 39.6 mL, *p* = 0.005) and better blood volume maintenance within the penumbra (CBV index: 0.7 vs. 0.5, *p* <0.001).

### 4.2 Representative case illustrations of the dynamic quantitative collateral approach

Figure 1 illustrates three patients with identical baseline Tan scores (Score 2) but markedly different clinical outcomes, which are accurately captured by the dynamic quantitative collateral approach. In the first case (A–C, a 64-year-old male with significant improvement), the high TCI (99%) was driven by a robust RCI, indicating rapid and effective collateral filling that minimized the final infarct core. In contrast, the second case (D–F, a 79-year-old female with no significant improvement) exhibited a much lower TCI (58%). Although some contrast arrival was noted in the LCI, the low RCI confirmed a failure of early rapid compensation, leading to the development of a large territorial infarct. Crucially, the third case (G–I, a 57-year-old female with no significant improvement) demonstrates the phenomenon of ineffective delayed collateral compensation. Despite a marked reduction in vascular deficit in the late venous phase (driving a high LCI of 70%), the early rapid compensation was inadequate (low RCI of 33%). This confirms that delayed collateral flow failed to rescue the ischemic tissue within the critical time window, resulting in a large irreversible infarct core and severe clinical deterioration (ΔNIHSS = −10). This case directly visualizes the physiological distinction between effective tissue salvage and hemodynamically ineffective delayed filling.

### 4.3 Criterion validity of the quantitative collateral indices

**Figure 2:**
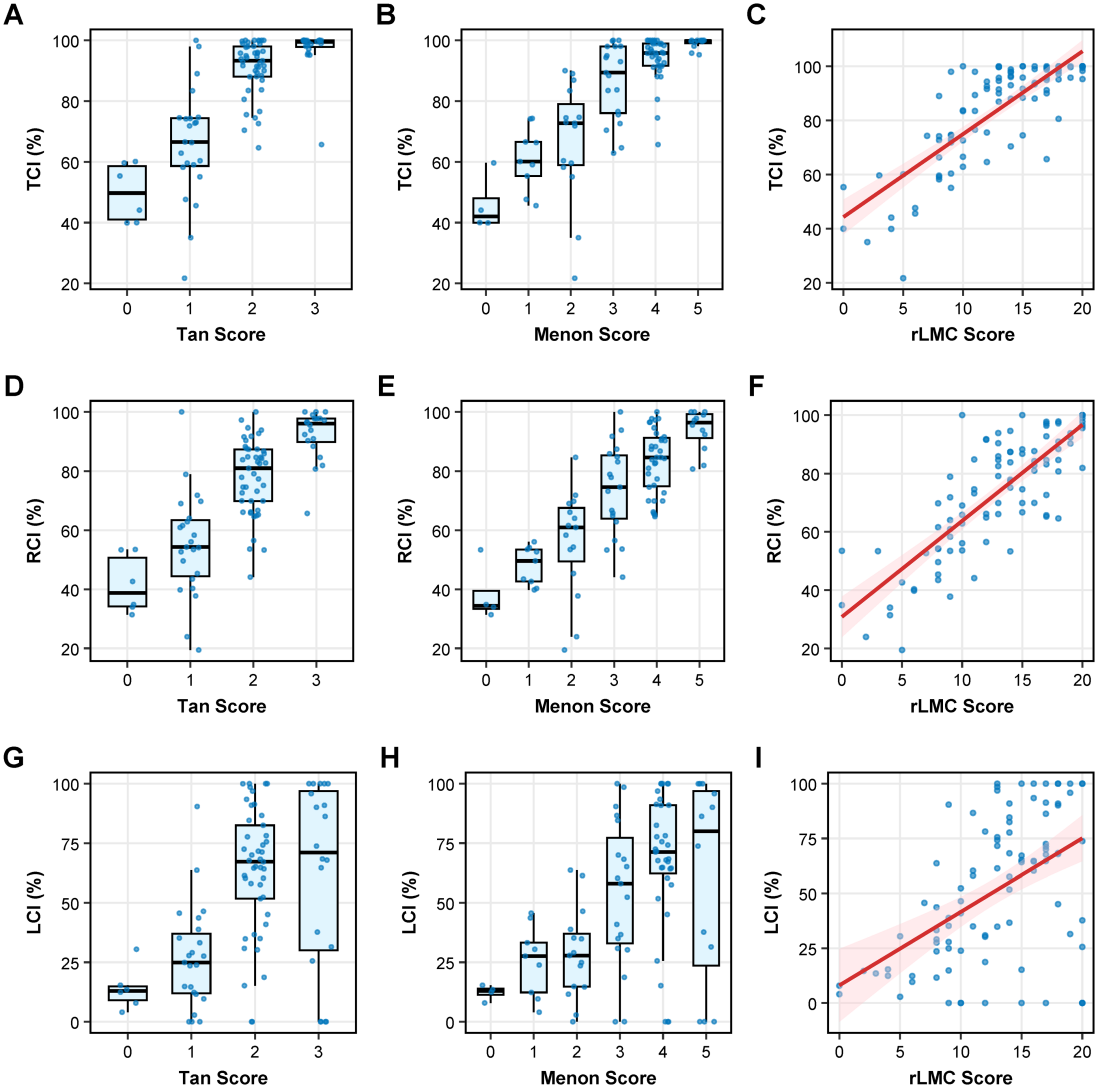
Correlation between the quantitative collateral indices (TCI, RCI, LCI) and traditional semi-quantitative collateral scores. **(A–C)** TCI demonstrates a strong, stepwise positive correlation with ascending grades of the Tan score **(A)**, Menon score **(B)**, and rLMC score **(C)** (Spearman *ρ* = 0.766, *p* < 0.001). **(D–F)** RCI exhibits similarly robust correlations with conventional visual scores (all ρ > 0.75, *p* < 0.001), validating its capacity to capture effective collateral filling. **(G–I)** In contrast, LCI shows only moderate associations with the Tan **(G)**, Menon **(H)**, and rLMC **(I)** scores (ρ = 0.467–0.476, *p* < 0.001). This divergence highlights that traditional scoring systems, which heavily rely on arterial and venous enhancement, may systematically underappreciate the delayed venous hemodynamics uniquely captured by LCI. TCI, Total Compensation Index; RCI, Rapid Compensation Index; LCI, Late Compensation Index; rLMC, regional Leptomeningeal Collateral.

We further assessed the criterion validity of the quantitative collateral indices by evaluating their correlations with validated expert visual grading schemes (Figure 2 and Table 2). Spearman rank correlation analysis revealed that TCI correlated strongly and positively with all three conventional grading systems. As shown in the boxplots, TCI increased incrementally with higher grades of both the single-phase Tan score and multiphase Menon score. The strongest agreement was observed between TCI and the time-resolved Menon score (*ρ* = 0.775, *p* < 0.001) while TCI also showed good consistency with the anatomically defined rLMC score (*ρ* = 0.766) and Tan score (*ρ* = 0.733).

**Table 2:** Criterion validity: correlations between RCI, LCI, and TCI and expert-level visual scores.

Variable	Tan score	Menon score	rLMC score
RCI	0.753 (0.637–0.844)	0.772 (0.666–0.845)	0.775 (0.651–0.858)
TCI	0.733 (0.602–0.837)	0.775 (0.667–0.850)	0.766 (0.640–0.860)
LCI	0.467 (0.269–0.654)	0.472 (0.274–0.644)	0.476 (0.272–0.657)

All correlation coefficients were statistically significant (*p* < 0.001). RCI, Rapid Compensation Index; TCI, Total Compensation Index; LCI, Late Compensation Index; rLMC, regional Leptomeningeal Collateral.

The RCI also showed strong correlations with all visual grading schemes (all *ρ* > 0.75, *p* < 0.001), supporting the ability of VVD to reliably quantify early collateral filling dynamics. By comparison, LCI exhibited only moderate associations with conventional visual scores (*ρ* = 0.467–0.476, all *p* < 0.001). This finding is physiologically reasonable, since LCI captures delayed venous hemodynamic features that are typically underappreciated by traditional scoring systems, which focus predominantly on arterial and early venous enhancement.

### 4.4 Inter-correlation among imaging and clinical parameters

**Figure 3:**
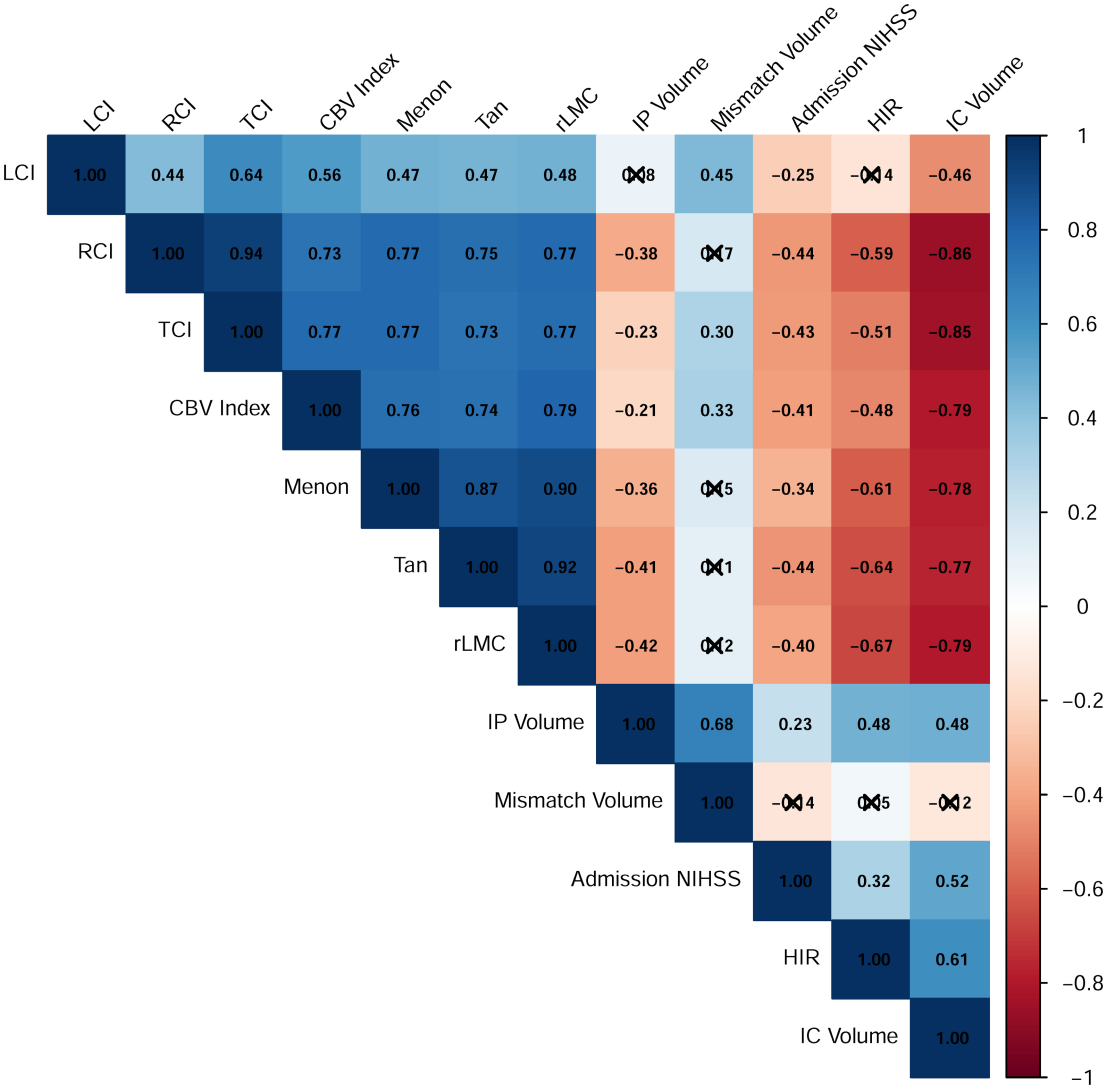
Correlation heatmap of clinical, traditional scoring, and the quantitative collateral indices. The heatmap visualizes Spearman’s rank correlation coefficients (*ρ*) across the quantitative collateral indices, visual collateral scores, tissue injury markers, and key clinical characteristics. Blue cells indicate positive correlations, while red cells indicate negative correlations, with color intensity reflecting the strength of the association. Crosses (×) denote non-significant correlations (*p* > 0.05). The matrix highlights a distinct clustering of collateral markers (top-left) exhibiting strong mutual inter-correlations, alongside their robust inverse associations with downstream tissue injury markers (e.g., Infarct Core Volume and HIR, far-right). TCI, Total Compensation Index; RCI, Rapid Compensation Index; LCI, Late Compensation Index; CBV, Cerebral Blood Volume; rLMC, regional Leptomeningeal Collateral; IP, Ischemic Penumbra; NIHSS, National Institutes of Health Stroke Scale; HIR, Hypoperfusion Intensity Ratio; IC, Infarct Core; CAD, Coronary Artery Disease; AF, Atrial Fibrillation.

The correlation heatmap reveals significant associations between the quantitative collateral indices and established clinical and perfusion parameters (Figure 3). The most striking finding was the strong inverse correlation between the RCI and TCI and the final infarct core volume (TCI: *ρ* = −0.85, *p* < 0.001; RCI: *ρ* = −0.86, *p* < 0.001). These correlations indicate that the dynamic morphological evolution of Vessel Volume Deficit (VVD) accurately reflects the extent of irreversible tissue injury.

Furthermore, TCI demonstrated a significant positive correlation with the CBV Index (*ρ* = 0.77, *p* < 0.001), indicating preserved blood volume within the penumbra, and a moderate inverse correlation with the HIR (*ρ* = −0.51, *p* < 0.001), a marker of severe hypoperfusion. Clinically, higher TCI levels were associated with lower admission NIHSS scores (*ρ* = −0.43, *p* < 0.001), suggesting that robust macroscopic collateral compensation translates to relative preservation of neurological function in the acute phase prior to intervention.

### 4.5 Univariate performance for predicting early neurological recovery

**Figure 4:**
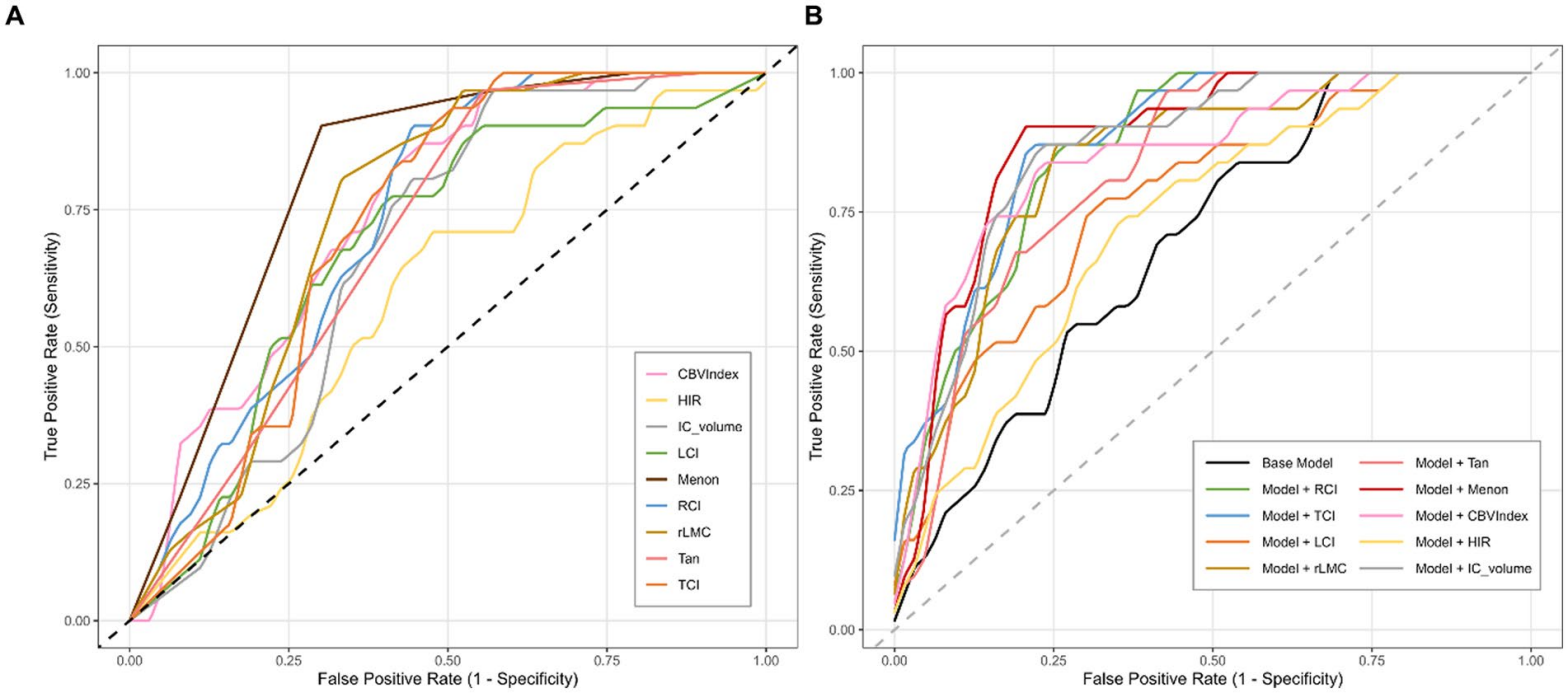
ROC curve analyses for predicting Early Neurological Recovery. **(A)** Univariate Analysis: ROC curves for individual imaging parameters. The Menon score demonstrated the highest discriminative ability among visual scores. Notably, while TCI and RCI showed robust predictive performance (surpassing the Tan score), LCI exhibited a marked attenuation in prognostic utility (AUC = 0.689). This divergence quantitatively visualizes the concept of “ineffective delayed compensation,” indicating that delayed contrast arrival fails to translate into effective clinical salvage. **(B)** Multivariable Analysis: ROC curves for logistic regression models. The Base Model (dashed line) includes Age, Admission NIHSS, Hypertension, and HbA1c. The addition of TCI (Model + TCI) yielded a substantial improvement in model performance, achieving an AUC comparable to the best-performing Model + Menon (DeLong test *p* = 0.313) (Table 3). Detailed AUC values, 95% confidence intervals, and AIC metrics for all models are presented in Table 3. ROC, Receiver Operating Characteristic; AUC, Area Under the Curve; TCI, Total Compensation Index; RCI, Rapid Compensation Index; LCI, Late Compensation Index; rLMC, regional Leptomeningeal Collateral; CBV, Cerebral Blood Volume; IC, Infarct Core; NIHSS, National Institutes of Health Stroke Scale; HbA1c, Glycated Hemoglobin.

Receiver Operating Characteristic (ROC) curve analysis was performed to evaluate the prognostic utility of each imaging biomarker for ENR (Figure 4 and Table 3). In univariate analysis, the traditional multiphase Menon score achieved the highest initial discrimination (AUC = 0.812), outperforming the single-phase Tan score (AUC = 0.704).

**Table 3:** Comparative performance of univariate and multivariable models for predicting early neurological recovery.

Variable	Univariate analysis			Multivariable analysis					
	OR (95% CI)	Uni. *P*	AUC (95% CI)	OR (95% CI)	Multi. *P*	AUC (95% CI)	AIC	*P* (vs. base)	*P* (vs. Menon)
Base model	–	–	–	–	–	0.694 (0.587–0.802)	120.35	–	<0.001
RCI	1.056 (1.024–1.088)	<0.001	0.734 (0.635–0.832)	1.091 (1.047–1.137)	<0.001	0.863 (0.791–0.934)	94.75	<0.001	0.546
TCI	1.086 (1.035–1.139)	<0.001	0.724 (0.624–0.823)	1.123 (1.060–1.189)	<0.001	0.878 (0.810–0.945)	90.71	<0.001	0.944
LCI	1.022 (1.007–1.037)	0.004	0.689 (0.577–0.801)	1.027 (1.009–1.045)	0.002	0.771 (0.672–0.870)	111.53	0.116	0.023
Tan	2.962 (1.532–5.726)	0.001	0.704 (0.611–0.796)	4.921 (2.104–11.513)	<0.001	0.828 (0.747–0.909)	103.61	0.006	0.03
rLMC	1.251 (1.107–1.413)	<0.001	0.745 (0.647–0.842)	1.363 (1.162–1.599)	<0.001	0.844 (0.762–0.925)	100.15	0.003	0.075
Menon	3.688 (1.969–6.907)	<0.001	0.812 (0.731–0.893)	4.725 (2.273–9.822)	<0.001	0.880 (0.809–0.950)	88.91	<0.001	Reference
CBV index	1.620 (1.248–2.103)	<0.001	0.750 (0.652–0.848)	1.997 (1.419–2.811)	<0.001	0.851 (0.768–0.934)	97.96	0.003	0.335
HIR	0.865 (0.714–1.048)	0.138	0.600 (0.482–0.719)	0.813 (0.657–1.006)	0.057	0.730 (0.625–0.834)	118.55	0.294	<0.001
IC volume	0.980 (0.968–0.993)	0.003	0.679 (0.573–0.786)	0.963 (0.944–0.983)	<0.001	0.865 (0.791–0.938)	93.53	<0.001	0.571

AIC, Akaike Information Criterion; AUC, Area Under the Curve; CBV, Cerebral Blood Volume; CI, Confidence Interval; HbA1c, Glycated Hemoglobin; HIR, Hypoperfusion Intensity Ratio; IC, Infarct Core; LCI, Late Compensation Index; Multi., Multivariable; NIHSS, National Institutes of Health Stroke Scale; OR, Odds Ratio; RCI, Rapid Compensation Index; rLMC, regional Leptomeningeal Collateral; TCI, Total Compensation Index; Uni., Univariate.

Among the quantitative collateral indices, both RCI and TCI demonstrated substantial predictive value (AUC = 0.734 and 0.724, respectively), superior to the static single-phase Tan score. In contrast, the HIR, an established surrogate of slow flow, demonstrated limited univariate prognostic utility for early recovery (OR = 0.865, 95% CI: 0.714–1.048; AUC = 0.600, *p* = 0.138). However, a marked divergence in predictive performance was observed across the temporal indices. While RCI was prognostic of clinical recovery, the LCI failed to demonstrate comparable capability (AUC = 0.689). This pronounced difference suggests that delayed contrast arrival in the late venous phase does not translate to effective clinical salvage compared to rapid early filling.

Based on the Youden index, the optimal threshold for TCI to predict ENR was identified as 72.9%, and the optimal RCI cutoff was 56.6%. A TCI value above 72.9% indicates overall sufficient collateral compensation and a correspondingly higher likelihood of early functional recovery. Although the univariate performance of the quantitative collateral indices trailed slightly behind expert multiphase scoring, their objective nature provides standardized prognostic information without the need for manual intervention.

### 4.6 Incremental predictive value in multivariable models

To evaluate the independent prognostic value of the imaging biomarkers, multivariable logistic regression models were constructed by adjusting for core clinical confounders, including Age, Admission NIHSS, Hypertension, HbA1c, and Occlusion Site (Base Model). The addition of any imaging metric significantly improved the predictive performance over the clinical Base Model alone.

DeLong tests revealed significant performance differences among the varying assessment tools. As expected, the model incorporating the expert multiphase Menon score (Model + Menon, AUC = 0.884) was significantly superior to the model relying solely on the single-phase Tan score (Model + Tan, AUC = 0.810, *p* = 0.012).

Specifically, multivariable binary logistic regression confirmed that TCI was a robust and independent predictor of ENR (OR = 1.123, 95% CI: 1.060–1.189, *p* < 0.001; AUC = 0.878), exhibiting a superior model fit (AIC = 90.71) compared to the single-phase Tan score model (OR = 4.921, 95% CI: 2.104–11.513, *p* < 0.001; AUC = 0.828, AIC = 103.61). Conversely, the addition of HIR to the Base Model yielded only marginal independent predictive value (OR = 0.813, 95% CI: 0.657–1.006, *p* = 0.057; AUC = 0.730, AIC = 118.55).

Crucially, the model integrating the TCI (Model + TCI) demonstrated robust predictive performance with an AUC of 0.858. Furthermore, Model + TCI yielded a superior model fit (AIC = 92.02) compared to the single-phase Tan model (AIC = 104.22). The DeLong test indicated no statistically significant difference in discriminative ability between the TCI model and the best-performing expert-level Menon model (*p* = 0.313). These multivariable findings confirm that the quantitative collateral indices provides independent prognostic information comparable to multiphase visual grading by experts, establishing its clinical effectiveness as an objective alternative.

### 4.7 Development and performance of the clinical prediction model

To further evaluate the incremental prognostic value of TCI for clinical practice, we constructed a multivariable prediction model. A “Base Model” was established incorporating the standard clinical predictors: Age, Admission NIHSS, and HbA1c. Subsequently, TCI was integrated to create the “Full Model.”

Performance analysis revealed that the inclusion of TCI significantly enhanced the model’s discriminative ability. The Full Model achieved an apparent C-index of 0.983. Given the potential for overfitting in finite samples, internal validation was performed via 1,000 bootstrap resamples, yielding a mean optimism of only 0.011. The resulting bias-corrected C-index remained robust at 0.972, confirming that TCI captures critical prognostic information with high stability.

**Figure 5:**
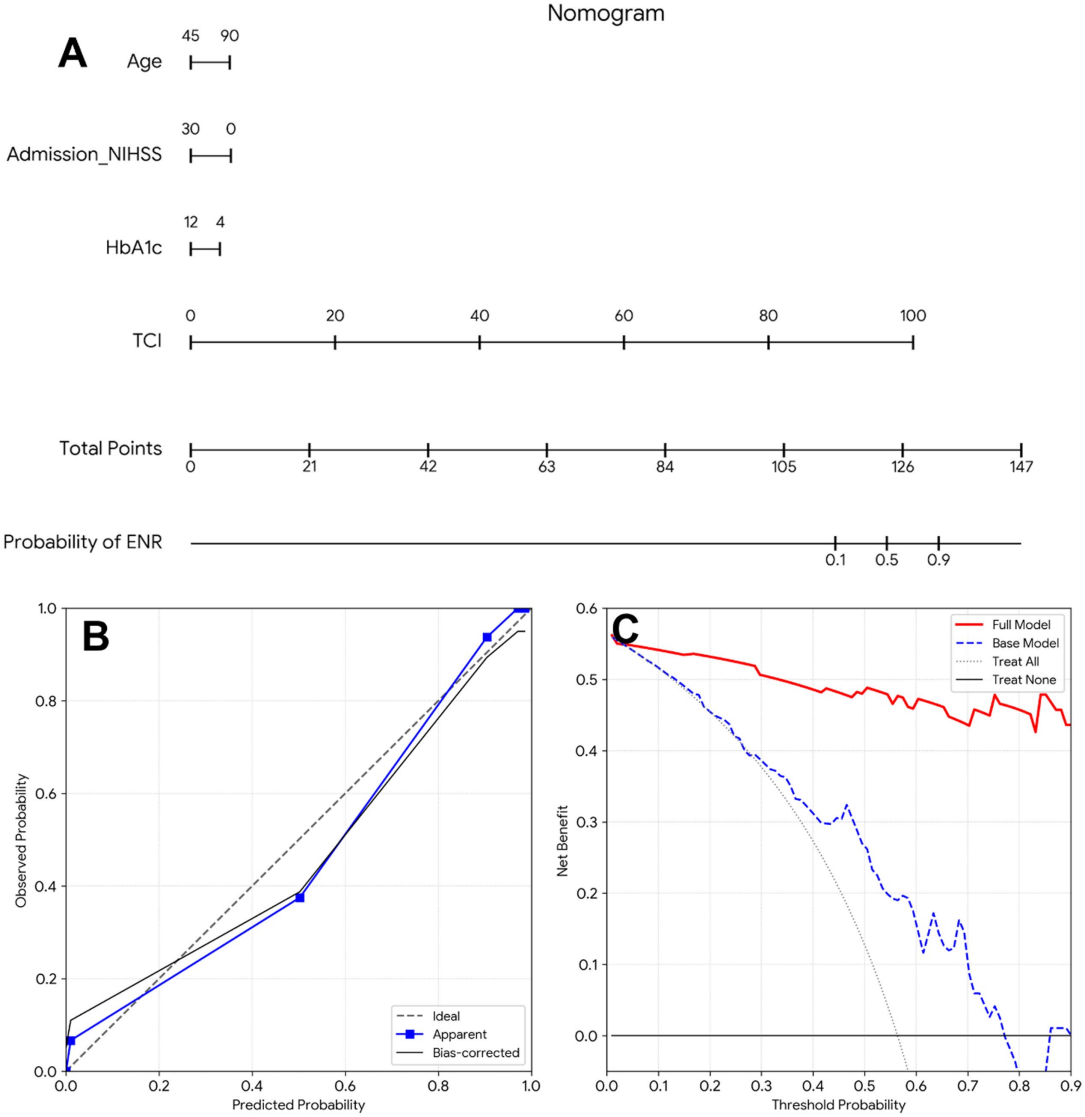
Construction and validation of the TCI-based clinical prediction nomogram. **(A)** Nomogram for predicting Early Neurological Recovery. The nomogram integrates Age, Admission NIHSS, HbA1c, and the TCI. To ensure visual clarity, the scales for Age, NIHSS, and HbA1c display only the minimum and maximum values, while the dominant predictor TCI is displayed with a full scale. Each variable corresponds to a point score on the top “Points” scale; the sum of these points corresponds to the predicted probability of ENR on the bottom scale. Note that the TCI axis spans the widest range, indicating its predominant contribution to the model. **(B)** Calibration Curve. The bias-corrected curve (black solid line) closely tracks the ideal diagonal line (gray dashed line), indicating excellent agreement between the nomogram-predicted probabilities and the actual observed outcomes. Internal validation via bootstrapping (*B* = 1,000) demonstrated high stability, with a bias-corrected C-index of 0.972. **(C)** DCA. The red line represents the Full Model (incorporating TCI), while the blue dashed line represents the Base Model (Age + NIHSS + HbA1c). The Full Model demonstrates a consistently higher net benefit across a wide range of threshold probabilities compared to the Base Model, treating all, or treating none, highlighting the incremental clinical utility of TCI. ENR, Early Neurological Recovery; NIHSS, National Institutes of Health Stroke Scale; HbA1c, Glycated Hemoglobin; TCI, Total Compensation Index; DCA, Decision Curve Analysis.

Based on the Full Model, a visual nomogram was developed to facilitate individualized risk stratification (Figure 5A). In the regression analysis, TCI exhibited the largest positive coefficient (0.307), followed by Age (0.037), while Admission NIHSS (−0.057) and HbA1c (−0.155) showed negative correlations. Correspondingly, in the nomogram, the scale for TCI is notably wider than those for Age, NIHSS, or HbA1c, identifying macroscopic dynamic collateral efficiency as the predominant determinant of early recovery in this cohort.

The calibration curve (Figure 5B) demonstrated excellent consistency between the nomogram-predicted probabilities and the actual clinical observations. Furthermore, Decision Curve Analysis (DCA) (Figure 5C) illustrated that the TCI-integrated model provided a superior net benefit across the entire range of reasonable threshold probabilities compared to the clinical Base Model. This suggests that incorporating TCI into acute decision-making protocols yields substantial clinical utility beyond relying solely on demographic and baseline severity indices.

## 5 Discussion

In this study, we introduced a deconvolution-free Dynamic Quantitative Collateral Approach based on 4D-CTA reconstructed from CTP source data (RCI, LCI, and TCI) to quantify collateral circulation and map the temporal evolution of ischemic tissue fate. Beyond simply automating collateral grading, our findings address two critical bottlenecks in acute stroke imaging: the mathematical vulnerability of standard CTP deconvolution algorithms in low-flow states, and the intrinsic inability of static visual scores to differentiate effective tissue salvage from delayed ineffective compensation. By demonstrating that the TCI offers independent predictive efficacy comparable to the expert-level multiphase Menon score and robustly inversely correlates with final infarct core volume, this study establishes a physiology-driven, objective alternative for precise acute stroke stratification.

Collateral circulation is a dynamic hemodynamic process (23). Traditional single-phase assessments often lose temporal information by relying on static images (24, 25). As d’Esterre et al. noted, time-resolved imaging is crucial to distinguish “no vessels” from “delayed filling vessels,” preventing the underestimation of collateral reserve (15). TCI addresses this by integrating data from the arterial to the late venous phase. The strong inverse correlation between TCI and infarct core volume ( ρ=−0.85 ) supports its pathophysiological validity: it quantifies not only the anatomical presence of collateral vessels but also their functional efficiency in salvaging brain tissue via retrograde perfusion.

Standard CTP remains the clinical cornerstone for penumbral evaluation (26, 27); however, its reliance on complex deconvolution kinetics renders it highly sensitive to severe tracer delay and low-flow hemodynamics. These conditions frequently induce mathematical regularization errors, leading to substantial volumetric overestimation of the ischemic core (28). To overcome these kinetic vulnerabilities, recent studies have increasingly explored deconvolution-free alternatives, such as utilizing deep learning to directly predict pixel-wise infarction from spatiotemporal 4D data (29). Concurrently, dynamic CTA segmentation strategies have been developed to distinctively separate arteriovenous phases (30). While these approaches successfully bypass singular value decomposition (SVD) artifacts to map macrovascular patency, they largely function as prognostic algorithms, lacking the physiological interpretability to explain microvascular failure, such as hemodynamically ineffective delayed filling.

To address this gap, in our study, the VVD is volumetrically quantified by extracting the vascular deficit mask via contralateral mirror comparison on dynamic MIPs. By bypassing traditional deconvolution modeling while maintaining physiological explainability, this approach directly captures the spatial-hemodynamic signature of contrast arrival. Consequently, it provides a methodologically robust and reproducible alternative for collateral volumetric assessment, particularly in critically ischemic patients where conventional CTP kinetics may fail to reflect true tissue viability (31).

While the expert-level Menon score showed the highest predictive accuracy in univariate analysis, TCI offers distinct clinical advantages. Traditional scoring is experience-dependent and time-consuming; TCI calculation is objective, eliminating human bias. Moreover, as a continuous variable (0–100%), TCI provides higher resolution than discrete grades, preserving hemodynamic detail. Our multivariable analysis confirmed that TCI provides independent predictive value comparable to multiphase scoring, supporting its non-inferiority as a standardized screening tool.

The clinical utility of TCI was further validated through nomogram and decision curve analyses. While advanced age, baseline NIHSS, and metabolic status (HbA1c) are established predictors of stroke outcome, our model demonstrates that in the acute phase of LVO, the hemodynamic status of collateral circulation outweighs these systemic factors. This is evident in the nomogram, where the TCI scale spans the widest range, contrasting with the relatively narrower contribution of age and HbA1c. Incorporating TCI into the diagnostic workflow facilitates hemodynamic risk stratification, ensuring that patients with robust collateral reserve are not undertreated solely based on advanced age or adverse metabolic profiles.

It is also important to acknowledge the inherent heterogeneity and lactate of large vessel occlusions in our cohort. The anatomical site of occlusion (e.g., ICA vs. MCA) directly influences baseline ischemic risk and available collateral pathways, as reflected by the significant differences in occlusion sites between our outcome groups. However, anatomical location alone does not fully dictate tissue fate. Our dynamic quantitative approach focuses on capturing the downstream physiological reality: whether the resultant collateral flow provides effective tissue salvage or merely represents delayed filling. By measuring the efficiency of vascular deficit resolution across temporal phases, functional indices like TCI and RCI intrinsically account for the anatomical variations in proximal occlusion sites.

By bypassing deconvolution modeling entirely, our framework directly captures the spatial-hemodynamic signature of contrast arrival. The quantitative collateral indices help elucidate the radiographic pattern of ineffective delayed compensation. Paradoxically, LCI was significantly higher in the clinical improvement group (71.3% vs. 36.7%, *p* = 0.003). This must be interpreted within the temporal hierarchy of collateral flow. In robust collateral networks, early and late filling mechanisms operate sequentially and efficiently. High RCI and LCI synergistically resolve the VVD, yielding a high TCI and favorable clinical outcomes. Conversely, a discordant hemodynamic pattern—low RCI paired with high LCI—reflects delayed collateral filling that is ultimately ineffective. Anatomically, contrast eventually fills the vascular bed. Hemodynamically, it arrives too late. We hypothesize that missing the critical ischemic tolerance window renders this delayed perfusion ineffective for penumbral salvage (32, 33). This strict time-dependence explains the diminished prognostic value of isolated LCI (AUC = 0.689) compared with RCI (AUC = 0.734). It also exposes a core flaw in static assessments such as the Tan score. Such visual metrics falsely equate delayed vessel patency with effective tissue perfusion. By capturing this temporal divergence, this dynamic approach demonstrate that comprehensive hemodynamic profiling should supersede simple anatomical visualization.

We noted an incidental finding of higher homocysteine and lactate levels in the significant improvement group. Although hyperhomocysteinemia is a known risk factor for stroke, its impact on acute phase collateral dynamics is unclear (34, 35). Given the relatively small sample size in the improvement group (*n* = 31) and the multiple comparisons performed, we cannot exclude the possibility that this is a Type I error or an incidental finding unrelated to collateral physiology. This association requires verification in larger cohorts.

Our study has several limitations. First, as a single-center retrospective analysis with a relatively modest sample size (*N* = 94), it carries an inherent risk of selection bias and limited statistical power in multivariable adjustments. Specifically, to isolate the independent prognostic value of baseline collaterals, our study design strictly included EVT patients who achieved complete recanalization (mTICI grade 3). While this stringent selection successfully eliminates the powerful confounding effect of varying reperfusion success, it limits the direct generalizability of our findings to patients experiencing partial or failed recanalization (mTICI < 3) in real-world practice. Furthermore, onset-to-recanalization time was not incorporated into our multivariable models due to its lack of baseline univariate significance (*p* = 0.077) and the finite number of outcome events (*N* = 31), prioritizing model stability over excessive covariate inclusion. By the same statistical rationale, while baseline treatment modalities (IVT, EVT, or bridging therapy) showed no significant difference between outcome groups, acute reperfusion therapies exert a profound individual influence on final tissue fate. The exclusion of these treatment variables from the multivariable model to prevent overfitting leaves a potential for residual confounding that must be acknowledged (36). However, this study was primarily designed as a pathophysiological proof-of-concept rather than a universally applicable clinical triage tool. Despite these confounding risks, the high bias-corrected C-index (0.972) observed during internal bootstrap validation indicates that the mechanistic relationship captured by TCI is highly stable and not merely an artifact of finite sample overfitting. Future prospective multicenter studies are required to generalize these thresholds for routine clinical application. Second, 90-day functional outcome (mRS) data were unavailable. While ENR directly reflects initial hemodynamic salvage and minimizes post-acute confounders, it cannot definitively predict 90-day functional independence. Therefore, our findings primarily indicate immediate tissue rescue rather than long-term outcomes. Third, while TCI characterizes macroscopic collateral compensation, it cannot account for the “no-reflow phenomenon” at the microcirculatory level, where tissue injury persists despite large vessel patency. Finally, the extraction of VVD relied on a specific proprietary AI algorithm, limiting external reproducibility. Additionally, the core voxel-wise mirror-subtraction logic inherently relies on the assumption of interhemispheric symmetry. Consequently, undetected anatomical variants (e.g., an incomplete Circle of Willis) or asymptomatic contralateral steno-occlusive disease could introduce volumetric bias into the calculations, although we attempted to mitigate this by excluding patients with prior large territorial infarctions. Subsequent research should explore generalizable open-source algorithms to achieve standardized cross-platform application.

In conclusion, this pilot study demonstrates the clinical feasibility and biological plausibility of a fully automated, deconvolution-free approach to dynamic collateral quantification. By tracking the temporal evolution of vessel volume deficits, the TCI successfully delineates the critical physiological boundary between effective tissue salvage and futile compensation. Despite sample size limitations, this method provides objective, physiology-driven prognostic accuracy comparable to expert multiphase consensus. Ultimately, this quantitative tool circumvents the mathematical vulnerabilities of standard perfusion imaging and the subjectivity of visual scores. It offers a standardized, robust imaging biomarker for evaluating early tissue salvage and immediate hemodynamic efficacy in acute stroke. Further large-scale trials are warranted to validate its predictive value for long-term functional outcomes.

## Data Availability

The raw data supporting the conclusions of this article will be made available by the authors, without undue reservation.

## Glossary

4D-CTA: four-dimensional computed tomography angiography
AIC: Akaike Information Criterion
AIS: acute ischemic stroke
AUC: Area Under the Curve
CBV: cerebral blood volume
CI: confidence interval
CT: computed tomography
CTP: computed tomography perfusion
DCA: decision curve analysis
DWI: diffusion-weighted imaging
ENR: early neurological recovery
EVT: endovascular thrombectomy
HbA1c: glycated hemoglobin
HIR: hypoperfusion intensity ratio
ICA: internal carotid artery
IQR: interquartile range
IVT: intravenous thrombolysis
LCI: late compensation index
LVO: large vessel occlusion
MCA: middle cerebral artery
MIP: maximum intensity projection
mRS: Modified Rankin Scale
NCCT: non-contrast computed tomography
NIHSS: national institutes of health stroke scale
OR: odds ratio
RCI: rapid compensation index
rCBF: relative cerebral blood flow
rLMC: regional leptomeningeal collateral
ROC: receiver operating characteristic
SD: standard deviation
SVD: singular value decomposition
TCI: total compensation index
TDC: time-density curve
VVD: vessel volume deficit
